# Intravesical dexmedetomidine instillation reduces postoperative catheter-related bladder discomfort in male patients under general anesthesia: a randomized controlled study

**DOI:** 10.1186/s12871-020-01189-2

**Published:** 2020-10-22

**Authors:** Hong Chen, Bin Wang, Qin Li, Juan Zhou, Rui Li, Ye Zhang

**Affiliations:** Department of Anesthesiology and Perioperative Medicine, The Second Affiliated Hospital of Anhui Medical University, 678# Furong Road, Hefei, Anhui Province China

**Keywords:** Dexmedetomidine, Intravesical instillation, Catheter-related bladder discomfort, General anesthesia

## Abstract

**Background:**

The catheter-related bladder discomfort (CRBD) of male patients is a common clinical problem, albeit lacking effective solutions. The present study aimed to investigate whether intravesical dexmedetomidine instillation alleviates the postoperative urinary discomfort in male patients with catheter under general anesthesia.

**Methods:**

This single-blinded, prospective, randomized study included a total of 167 male patients American Society of Anesthesiologists (ASA) physical status I-II scheduled for surgery under general anesthesia were allocated to two groups: 84 in the dexmedetomidine group and 83 in the control group. Dexmedetomidine group patients received intravesical instillation of the drug 0.5 μg/kg and normal saline 20 mL, while the control group received intravesical instillation of 20 mL normal saline. The catheter was clamped for 30 min after intravesical instillation for all patients. CRBD scores and urethra pain numerical rating scale (NRS) scores were measured at admittance to post-anesthesia care unit (PACU) (T0), intravesical instillation (T1), 30 min (T2), 60 min (T3), 2 h (T4) after intravesical instillation, discharged from PACU (T5), and 6 h (T6) and 24 h (T7) after the operation. Patient satisfaction at discharge from PACU and 24 h post-operation were compared between the two groups.

**Results:**

CRBD scores and urethra pain NRS scores after 30 min of intravesical dexmedetomidine instillation to 24 h post-operation were significantly lower than the control group (*p* < 0.001), and patient satisfaction was higher at discharge from PACU and 24 h post-operation (*p* < 0.001). No differences were detected in Steward score out of PACU (*p* = 0.213) and from the time of the end of operation to fully awake (*p* = 0.417).

**Conclusion:**

Intravesical dexmedetomidine instillation reduces postoperative urinary discomfort and urethra pain and improves satisfaction in male patients under general anesthesia.

**Trial registration:**

Chinese Clinical Trial Registry (No. ChiCTR1800016429), date of registration 1st June 2018.

## Background

Catheter-related bladder discomfort (CRBD) after the operation is a common adverse reaction; however, many surgery patients need an indwelling catheter during or after the operation. The incidence of CRBD is 47–90% [1, 2], and catheter maladjustment is common, especially in male patients [3, 4]. Reportedly, 27–55% of the patients experience moderate or severe catheter-related bladder discomfort symptoms in the post-anesthesia care unit (PACU) [3, 5]. CRBD leads to restlessness, delirium, decreased satisfaction, and a rise in postoperative complications, such as incision rupture, bleeding, hemodynamic instability, the severity of coronary heart disease [1]. Thus, how to relieve postoperative CRBD, reduce the incidence of related complications, improve patient satisfaction, and shorten the time of anesthesia recovery observation has become needs to be resolved urgently in clinical practice [6–9].

Dexmedetomidine is a type of high selective adrenergic α-2 receptor agonist, which has the effects of sedation, analgesia, and anti-anxiety. Several studies have confirmed that dexmedetomidine can reduce the incidence of postoperative agitation and delirium [10–14]. Moreover, dexmedetomidine might reduce bladder contractility via α-2 receptor agonism, M3 muscarinic receptor antagonism [15–17], and the incidence and severity of catheter-related bladder discomfort after general anesthesia [16, 18–21]. These studies were based on the intravenous administration of dexmedetomidine, which increases the risk of arrhythmia [22].

A large number of studies [23–25] showed that intravesical instillation is an effective way of drug administration, which exerts an obvious effect on the treatment of bladder-related diseases. Concurrently, the intravesical instillation of drugs can reduce the systemic response. However, whether dexmedetomidine can be used by intravesical instillation to reduce CRBD and improve the tolerance to indwelling catheter has not yet been reported. Thus, the present study aimed to investigate whether intravesical dexmedetomidine instillation can alleviate the postoperative urinary discomfort in male patients with general anesthesia.

## Methods

### Study design

This single-center, single-blinded, prospective, randomized study was approved by the Institutional Review Board and Hospital Research Ethics Committee of the Second Affiliated Hospital of Anhui Medical University [No. PJ-YX2018–004 (F2)]. The protocol of the study was registered in the Chinese Clinical Trial Registry (No. ChiCTR1800016429) and executed in accordance with the CONSORT checklist. Each patient provided written informed consent before participation in the study. Patients can withdraw from the study at any time according to their wishes. All patients were enrolled between June 2018 and April 2019. The inclusion criteria were as follows: male patients aged 18–70 years and American Society of Anesthesiologists (ASA) physical status I-II undergoing elective surgery, scheduled general anesthesia, and intraoperative catheter insertion. The exclusion criteria were as follows: urology patients, end-stage renal disease, pathological obesity, central nervous system dysfunction, chronic pain, cerebral infarction, mental disorder of consciousness, change in surgical and anesthesia plans, without CRBD when admitted to PACU. In this single-blinded study, patients were blinded to the group allocation. However, it was not blinded to anesthesiologists, PACU nurses, surgeons and ward nurses because this information is important for perioperative management of patients.

### Sample size

PASS 11.0 software was used to compare the mean of two independent samples. According to the pre-experiment CRBD score, the mean value of the control group was 2.25, and the standard deviation was 0.66. The mean value of the dexmedetomidine group was 1.9 and the standard deviation was 0.67. Set α as 0.05, β as 0.9, using bilateral test, the sample size of each group is 75. Increase the sample size 20% to prevent the sample drop-out, so we chose 90 patients in each group.

### Patient randomization

Male patients with CRBD into PACU were randomly divided into two groups with an allocation ratio of 1:1 according to the computerized randomization table in a blinded manner. Random numbers to each patient while the nurses collected postoperative data from the patients.

### Anesthesia application

The surgery and anesthesia program of the patient was similar to that of the other patients. Electrocardiography (ECG), peripheral oxygen saturation (SpO_2_), non-invasive blood pressure (NBP), and respiratory rate (RR) were monitored routinely after the patients were admitted to the operating room. The vein channel was established with a 22-gauge indwelling needle. Oxygen was inhaled by mask (oxygen flow rate was 4–5 L/min). Midazolam 0.025 mg/kg, sufentanil 2–4 μg/kg, etomidate 1–2 mg/kg, and rocuronium 0.9 mg/kg were injected intravenously for anesthesia induction. After intubation, a ureteral catheter was used for catheterization, followed by anesthesia maintenance propofol 2–4 mg/kg/h, remifentanil 10–20 μg/kg/h, and continued addition of cisatracurium to maintain muscle relaxation. At the end of the operation, the muscle relaxation and consciousness of the patient were restored, the tracheal tube was removed and sent to PACU for observation.

### Catheterization

After induction of anesthesia, 16F latex ureteral catheter (Huaxing Medical Equipment Co., Ltd., China) was used for all participants. Catheterization was performed by surgeons with more than 5 years experience. The operation process must be as gentle as possible, and the whole process was sterile. Before catheterization, paraffin oil fully lubricated the catheter. After the successful placement of the catheter, 10 mL of normal saline was injected into the cuff balloon to prevent catheter slippage. After catheterization, the catheter was fixed on the inside of the thigh to prevent urinary tract injury caused by catheter pulling.

### Interventions

As a safe and widely used drug, intravesical dexmedetomidine instillation method has been approved by the Institutional Review Board and Hospital Research Ethics Committee of the Second Affiliated Hospital of Anhui Medical University. In the dexmedetomidine group, 0.5 μg/kg dexmedetomidine was solubilized in 20 mL normal saline infused from the ureteral catheter to bladder for intravesical dexmedetomidine instillation. In the control group, 20 mL of normal saline was infused from the ureteral catheter to the bladder. After instillation, the ureteral catheter was clipped for 30 min and then unclipped.

### Assessments

The primary outcome endpoint was CRBD score, and the second outcome endpoint was urethra pain NRS score and patient’s satisfaction. The duration of anesthesia, the time length of operation, the time from the end of the operation to full consciousness, and patient characteristics were recorded. Mean arterial pressure (MAP), heart rate (HR), RR, SpO_2_, CRBD score, and urethra pain NRS score were recorded when the patient was sent to PACU (T0), the time of intravesical instillation with dexmedetomidine or normal saline (T1), 30 min after intravesical instillation (T2), 1 h after intravesical instillation (T3), 2 h after intravesical instillation (T4), the time point of leaving PACU (T5), 6 h after operation (T6), and 24 h after opertaion (T7). The NRS score of urethra pain and the complications after intravesical instillation were also recorded. The patient satisfaction score were recorded when leaving PACU and 24 h after the operation. The NRS score was used for the assessment of urethra pain in both groups. CRBD score: 0 point, patients have no discomfort at all; 1 point, patients have slight discomfort, only when asked to show discomfort; 2 points, patients have moderate discomfort, frequency of urination, the urgency of urination, feeling of lower abdominal distension, which is not easy to bear; 3 points, the patient had severe discomfort, intolerable distension, urethral pain, frequent urination with strong restlessness, and needed to be removed. Ramsay score: 1 point, the patient is restless and fidgety; 2 points, the patient is quiet and cooperative; 3 points, the patient is sleepy and can follow the instructions; 4 points, the patient is in a sleep state and can wake up; 5 points, the respiratory response of the patient is slow; 6 points, the patient is deep asleep and has no response to stimulation. Patient satisfaction score is consisting of integers from 1 point to 5 points: 1 point means dissatisfied and 5 points mean very satisfied. The urethra pain NRS scores consists of integers from 0 to 10 points; 0 point means no urethra pain, and 10 points indicate intense urethra pain. Subsequently, the patients selected an integer to describe the intensity of their urethra pain while using a ureteral catheter.

### Statistical analysis

SPSS software (version 22.0, Chicago, USA) was used for statistical analysis. The age, weight, blood, and other measurement data of patients were presented as mean ± standard deviation. The ASA classification data were expressed as counts. Student’s *t*-test or the Mann-Whitney U test was used for continuous variables, such as age and weight. The χ^2^ or Fisher’s exact tests were assessed for categorical variables, such as ASA grade and patient satisfaction score. ANOVA was used for the comparison of MAP, HR, SpO_2_, and other data at different time points. *p*-value < 0.05 was accepted as statistically significant.

## Results

### Study demographics

**Fig. 1:**
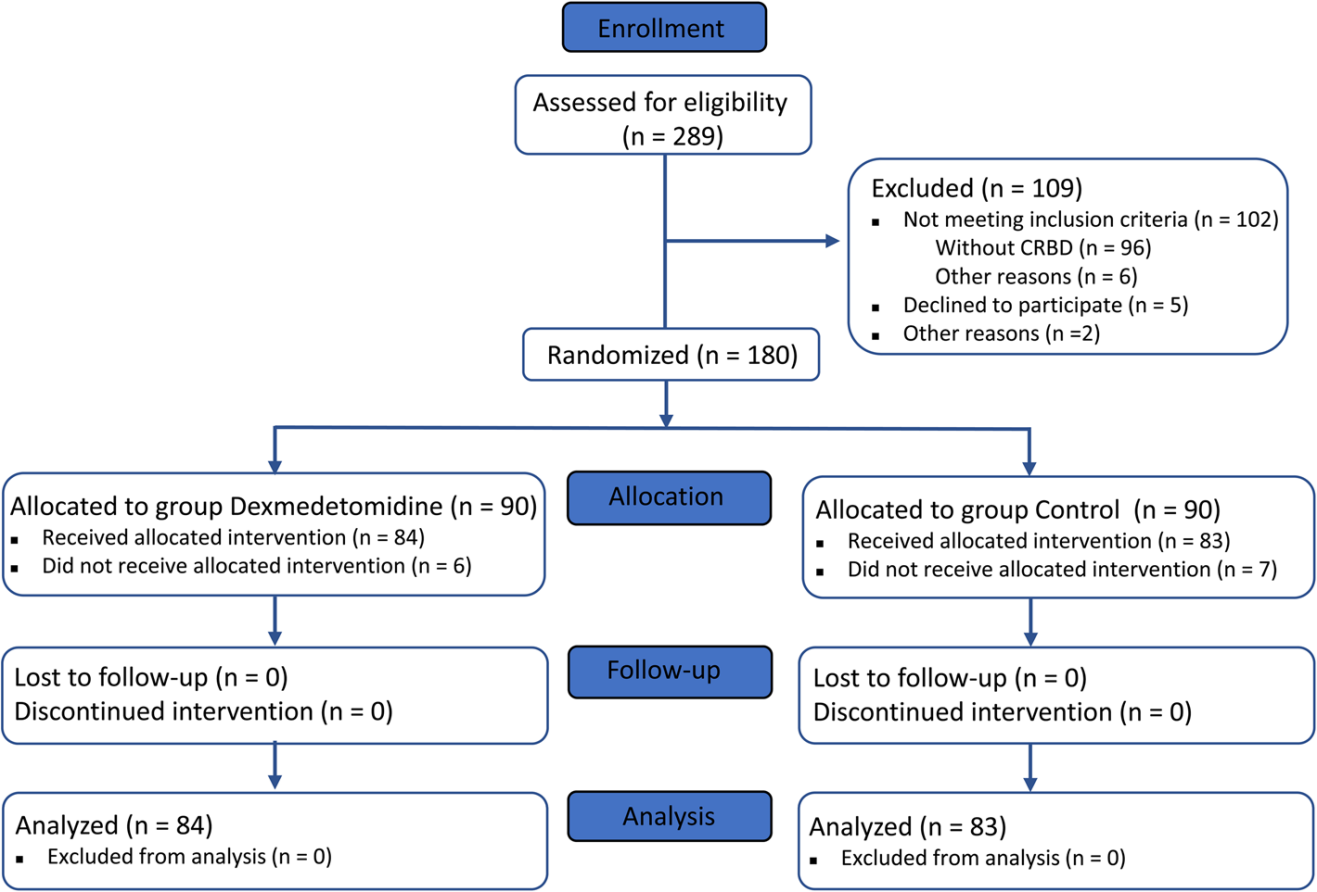
Schematic of the study with a CONSORT diagram. CRBD, catheter-related bladder discomfort

A total of 289 male patients were screened in this study, of which, 109 cases were excluded; among them, 96 did not present CRBD, 6 were not meeting the other inculsion criteria, 5 refused to participate in the study, and 2 were excluded for other reasons. A total of 180 male patients with CRBD were randomly and equally divided into both groups when into PACU. Six cases in the dexmedetomidine group and 7 cases in the control group did not complete the experiment because transfer to intensive care unit (2 cases), delirium (10 cases), or catheter falls off (1 cases). None of the patients were lost follow-up. Finally, 84 patients in the dexmedetomidine group and 83 patients in the control group were included in the analysis (Fig. 1). The incidence of CRBD was 65.2%.

No significant difference was detected between the two groups in age, weight, ASA grade, duration of operation, duration of anesthesia, Steward’s score when leaving PACU, the time length of operation end to fully awake, catheter removal time (Table 1) and type of operation. There was no significant difference was observed in HR, RR, MAP, and SpO_2_ in the two groups from T0 to T7 (Table 2), as well as no complications, occurred in either of the groups. Systematic pain NRS score decreased at T4.

**Table 1:** Descriptive variables of the control group and dexmedetomidine group

	Control (*n* = 83)	Dexmedetomidine (*n* = 84)	*p-*value
Age (year)	51.2 ± 13.2	54.7 ± 12.5	0.076
Weight (kg)	68.1 ± 10.5	68.3 ± 11.1	0.919
ASA grade (I/II)	7/76	9/75	0.617
Time length of operation (min)	146.6 ± 70.8	160.2 ± 62.7	0.193
Time length of anesthesia (min)	175.2 ± 72.2	190.2 ± 67.6	0.122
Steward score out of PACU	5.98 ± 0.15	5.95 ± 0.21	0.213
Time length of operation end to fully awake (min)	13.6 ± 6.5	14.9 ± 7.3	0.417
Ureteral catheter removal time (6–12 h/12–24 h/more than 24 h after operation)	12/46/25	9/38/37	0.173
Operation type (general surgery/thoracic surgery/orthopedics/otolaryngology/stomatology/ plastic surgery)	33/14/31/5/0/0	31/20/23/7/1/2	0.343
The number of patients receiving postoperative analgesia treatment	22 (26.5%)	21 (25.0%)	0.482

Values are presented as mean (standard deviation) or counts

*ASA* American society of anesthesiologists

**Table 2:** Patients vital signs of group Dexmedetomidine (*n* = 84) and group Control (*n* = 83) at different time points

	Group	T0	T1	T2	T3	T4	T5	T6	T7
HR	Control	76.6 ± 15.4	75.9 ± 15.0	77.4 ± 14.7	79.0 ± 14.0^##^	80.1 ± 14.3^##^	80.9 ± 14.2^##^	80.2 ± 12.4^##^	79.7 ± 12.5^#^
	Dexmedetomidine	75.6 ± 15.2	75.4 ± 15.1	75.8 ± 14.5	77.8 ± 13.9^#^	79.1 ± 14.3^##^	79.1 ± 14.1^#^	78.7 ± 11.0^#^	79.7 ± 12.5
RR	Control	18.8 ± 1.1	18.8 ± 1.1	18.9 ± 1.3	19.2 ± 1.0^##^	19.1 ± 1.0	19.1 ± 0.9^#^	19.1 ± 0.9^#^	18.9 ± 0.9
	Dexmedetomidine	18.8 ± 1.5	18.7 ± 1.2	18.5 ± 1.2	18.5 ± 1.4	18.7 ± 1.1	19.0 ± 1.0	18.7 ± 0.8	18.8 ± 0.8
MAP	Control	98.9 ± 16.1	97.9 ± 14.4	97.8 ± 14.5	97.3 ± 14.4	95.6 ± 14.4^#^	94.3 ± 12.7^##^	91.4 ± 11.5^##^	89.6 ± 10.5^##^
	Dexmedetomidine	98.4 ± 15.2	97.7 ± 15.5	95.4 ± 14.3^##^	94.8 ± 14.2^##^	94.1 ± 12.8^##^	92.8 ± 12.3^##^	91.5 ± 10.6^##^	89.6 ± 8.6^##^
SPO_2_	Control	98.7 ± 1.1	98.9 ± 1.2	98.6 ± 1.3	98.7 ± 1.2	98.7 ± 1.1	98.6 ± 1.2	98.3 ± 1.0^##^	98.3 ± 0.7^##^
	Dexmedetomidine	98.7 ± 1.8	98.9 ± 1.4	98.8 ± 1.4	98.8 ± 1.4	98.8 ± 1.1	98.8 ± 1.1	98.4 ± 0.9	98.3 ± 0.7
Systemic pain NRS score	Control	3.2 ± 1.2	3.1 ± 1.1	2.5 ± 0.7^##^	2.4 ± 0.7^##^	2.3 ± 0.7^##^	2.2 ± 0.7^##^	2.3 ± 1.0^##^	1.4 ± 0.9^##^
	Dexmedetomidine	3.0 ± 1.1	2.9 ± 1.0	2.5 ± 0.8^##^	2.2 ± 0.7^##^	2.1 ± 0.6^##*^	2.0 ± 0.6^##^	2.2 ± 1.4^##^	1.3 ± 0.8^##^

^*^p-value < 0.05 compared to the same time of Group Control (^*^p-value = 0.021)

^#^*p-*value < 0.05 compared to T0; ^##^*p-*value < 0.01 compared to T0

Values are presented as mean ± standard deviation

*MAP* Mean arterial pressure, *HR* Heart rate, *RR* Respiratory rate, *SpO*_*2*_ Pulse oximetry. Time course, the time point of the patient sent to PACU (T0), intravesical instillation (T1), 30 min after intravesical instillation (T2), 1 h after intravesical instillation (T3), 2 h after intravesical instillation (T4), leaving PACU (T5), 6 h after the operation (T6), 24 h after the operation (T7)

### CRBD and urethra pain NRS scores

**Fig. 2:**
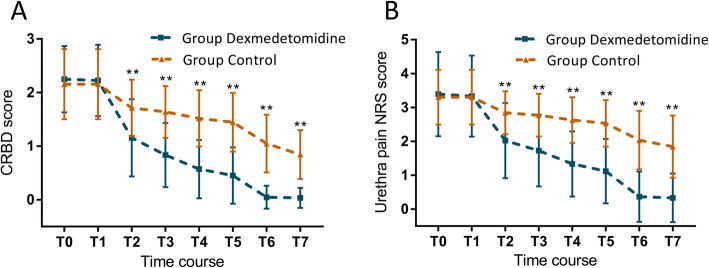
Bladder stimulation scale (Fig. 2a) and urethra pain NRS score (Fig. 2b) in dexmedetomidine and control groups at various time points. CRBD, catheter-related bladder discomfort. Time course, the time point at which the patient sent to PACU (T0), intravesical instillation (T1), 30 min after intravesical instillation (T2); 1 h after intravesical instillation (T3), 2 h after intravesical instillation (T4), leaving PACU (T5), 6 h after operation (T6), 24 h after operation (T7); **p < 0.001

Compared to the control group, the CRBD in the dexmedetomidine group was significantly improved at T3, T4, T5, T6, and T7 (*p* < 0.001) (Fig. 2a), while the urethra pain NRS scores of patients was significantly decreased (*p* < 0.001) (Fig. 2b).

### Patient’s satisfaction

**Fig. 3:**
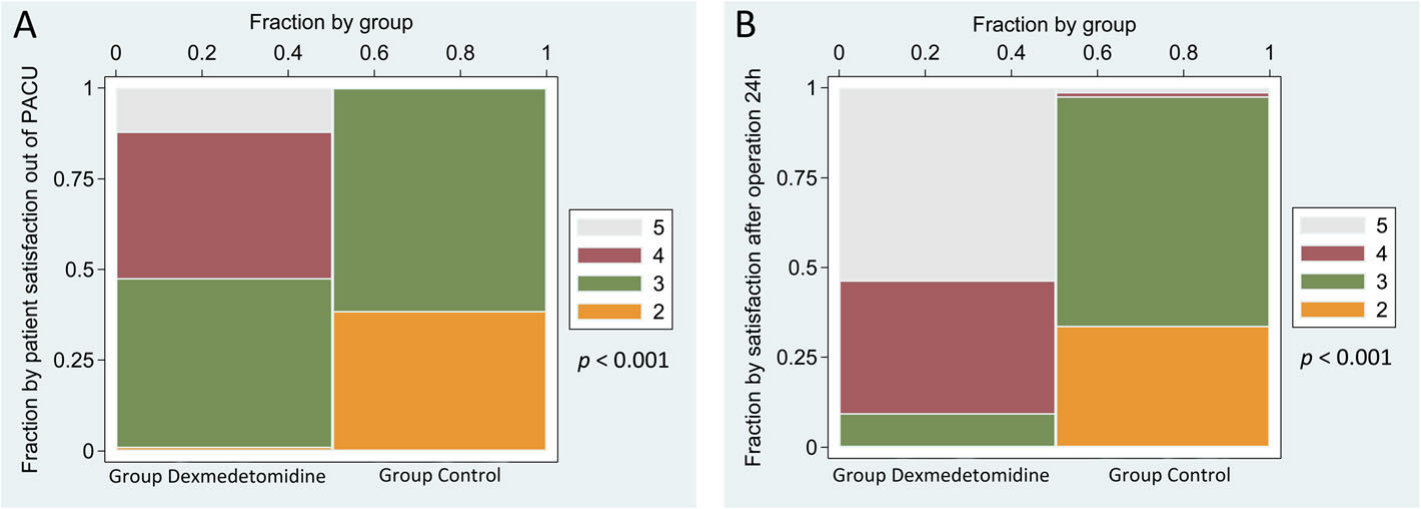
Spine plot of patient satisfaction score out of PACU (Fig. 3a) and 24 h after the operation (Fig. 3b) in dexmedetomidine and control groups. Patient satisfaction score: 5 points, very satisfied; 4 points, satisfied; 3 points, relatively satisfied; 2 points, basically satisfied; 1 point, dissatisfied

Compared to the control group, the patient’s satisfaction in the dexmedetomidine group in PACU (Fig. 3a) and 24 h post-operation (Fig. 3b) increased significantly (*p* < 0.001).

## Discussion

In the present study, we observed that 0.5 μg/kg dexmedetomidine intravesical instillation can significantly reduce the symptoms of postoperative catheter-related bladder discomfort and the urethral pain caused by catheter in male patients who received general anesthesia, and consequently, their satisfaction was improved. The improvement of these symptoms can sustain from 0.5–24 h after intravesical dexmedetomidine instillation.

CRBD is common in PACU, especially male patients [26]. Therefore, in this study, we included male patients as the study subject, and the incidence of CRBD was 65.2%, which was consistent with that reported previously [1, 2, 26]. The high incidence of CRBD in male patients might be related to the anatomical characteristics, such as the long urethra and large catheter [26]. Because several urological operations need to operate on the urethra, which markedly impacts this study, and the degree of impact is different, so this study was not included in the urological patients. In the clinical practice, to deal with bladder catheter pain, we often scribble lidocaine cream at the urethral orifice, meanwhile intravenous analgesic drugs, such as fentanyl, flurbiprofen axetil could also be used, but the effect is not ideal.

In this study, we observed that intravesical dexmedetomidine instillation can significantly reduce the symptoms of postoperative CRBD according to the following underlying mechanism. Alpha 2-adrenoceptor, i.e., the α2A-subtype, is expressed in the bladder, urethra, and prostate. The intra-arterial administration of an α-2 agonist reduced the micturition pressure, bladder capacity, and micturition volume [27, 28]. dexmedetomidine is a high selective adrenergic α-2 receptor agonist which may reduce the micturition pressure, bladder capacity, and micturition volume. There are several muscarinic receptors in bladder epithelium and efferent nerves, including M2 and M3. The M3 receptor is mainly responsible for bladder contraction [29]. The catheter can stimulate the afferent nerves of the bladder to release acetylcholine, which leads to the contraction of detrusor mediated by muscarinic receptors. Therefore, muscarinic antagonists alleviate CRBD in different degrees [1]. Some studies showed that dexmedetomidine might reduce bladder contractility via α-2 receptor and M3 muscarinic receptor antagonism [16, 17]. On the other hand, catheter stimulation can cause inflammation and increase prostaglandin secretion, which is one of the plausible reasons for CRBD [30]. Therefore, some anti-inflammatory drugs can also alleviate CRBD. Another study showed that dexmedetomidine reduces the release of prostaglandins of inflammation, and hence, relieves CRBD [31]. In addition, dexmedetomidine exerts a sedative effect and relieves CRBD [10]. Another underlying mechanism is that after intravesical instillation, dexmedetomidine can be absorbed from bladder and play a systemically role of sedation, analgesia and anti-inflammatory, which need to be further verified.

In the current study, dexmedetomidine plays a role in intravesical instillation. The off-label method of dexmedetomidine is often used in clinical research, which has proved to be safe and effective. For example, dexmedetomidine is safely used in subarachnoid and epidural [32], neuraxial [33] and for children intranasal [34]. As a safe and widely used drug, intravesical dexmedetomidine instillation method has been approved by the Institutional Review Board and Hospital Research Ethics Committee. Several studies [23–25] showed that intravesical instillation is an effective way of administration of drugs, which had an obvious effect on the treatment of bladder-related diseases and reduce the systemic response. For instance, invasive immunotherapy, chemotherapy and chemohyperthermia for bladder cancer [35, 36], intragastric thermal gelatin matrix implantation for intractable hematuria [37], intragastric gentamicin for recurrent urinary tract infections treatment [38]. Dexmedetomidine is well absorbed through the mucous membrane. Iirola et al. [39] reported that peak plasma concentrations of dexmedetomidine were 38 min after intranasal administration, and the pharmacological effects were similar to the intravenous administration but with a later onset time. In the current study, dexmedetomidine was able to work through the bladder mucosa, with a significant effect at half an hour after administration. Even so, intravesical dexmedetomidine instillation has potential risk of bladder dysfunction such as urinary retention through a local α2-stimulating effect, which should be closely pay attention in clinical practice.

In this study, intravesical dexmedetomidine instillation alleviates the pain caused by catheter while in situ and on removal. The main causes of the pain during catheter in situ were as follows: the material and size of the catheter, the traction of the catheter drainage bag, the urethral discomfort, the stimulation of the bladder wall by the catheter, the obstruction of the catheter, catheter blockage, the hemorrhagic pseudopolyps, the fear of the catheter, and the psychological rejection [40]. In the present study, all patients were observed and nursed closely, and the material and size of the catheter were identical, and no catheter drainage bag traction, catheter obstruction, hemorrhagic pseudopolyps were observed. Therefore, we speculated that the main reason for the difference in the urethral pain between the two groups was the tolerance of catheter stimulation of the bladder wall and the difference in the fear and psychological rejection of the catheter. Dexmedetomidine is a solution to bladder irritation and psychological maladjustment of patients, thereby reducing the catheter-induced urethral pain. Systematic pain NRS score decreased at T4, and there was no significant change at other time points. The possible reason is that dextromethorphan was absorbed by bladder and played a systemic role, which need further study.

Patients’ satisfaction at the time point out of PACU and 24 h after the operation was significantly improved after intravesical dexmedetomidine instillation because there were reductions in CRBD and catheter-induced urethral pain and patient satisfaction is closely related to the postoperative outcomes [41]. The improvement in the patients’ satisfaction might reduce their CRBD and urethral pain.

No complications were detected in the control and experimental groups. Since the sample size of this study is small, and the patients selected are ASA I-II, their basic conditions are well. Clinically, we will encounter CRBD to aggravate the condition of patients with coronary heart disease, and dexmedetomidine might also lead to arrhythmia and other risks. What is more, intravesical dexmedetomidine instillation perhaps could prolong bladder dysfunction through a local α2-stimulating effect and dexmedetomidine still reduces CRBD after 24 h. There was no urinary retention or re-catheterisation complication in dexmedetomidine group. However, only 9 ureteral catheters were removed at 6 to 12 h after operation, and 38 catheters were removed at 12 to 24 h in this study. The safety of the intravesical dexmedetomidine instillation need further clinical validation.

Nevertheless, the present study has some limitations. The number of cases is small as only 167 patients were included in this single-center study. In the future, large sample and multi-center clinical verification is essential. The patients included in this study were male patients with catheter placement under general anesthesia. The type of operation is not uniform, and the duration of operation is varied. This study did not limit the factors such as midazolam and operation time, and did not make further subgroup analysis. Further subgroup study can be carried out after expanding the sample size in the future. Prolonged follow-up for bladder dysfunction was not implemented. The safety of intravesical dexmedetomidine instillation should be further studied. It is not sure if systemic dexmedetomidine has the same effect for CRBD, further research should include an arm with intravenous dose of dexmedetomidine. All the patients included in this study were ASA I-II patients with elective surgery, and the basic condition of the patients was good. Also, critical patients have not been analyzed previously. Moreover, the effect of different doses of dexmedetomidine on CRBD was not assessed in this study. Ten milliliter normal saline was injected into the cuff balloon of catheter for all paitents to prevent slippage in our research. This may also be a potential cause of bladder wall irritation. It is regretful that we did not follow up with further research on the effect of reducing the balloon volume for reducing CRBD.

## Conclusions

Dexmedetomidine 0.5 μg/kg intravesical instillation reduces postoperative urinary discomfort and urethra pain caused by catheter in male patients under general anesthesia and improves patient satisfaction after the operation.

## Data Availability

The datasets used and analyzed during this current study are available from the corresponding author on reasonable request.

## Abbreviations

CRBD: Catheter-related bladder discomfort
ASA: American Society of Anesthesiologists
NRS: Numerical rating scale
PACU: Post-anesthesia care unit
ECG: Electrocardiography
SpO_2_: Peripheral oxygen saturation
NBP: Non-invasive blood pressure
RR: Respiratory rate
MAP: Mean arterial pressure
HR: Heart rate
